# Testosterone levels at diagnosis: A key predictor of overall survival among patients with prostate cancer

**DOI:** 10.1002/bco2.484

**Published:** 2025-02-17

**Authors:** Ilkka Jussila, Juha P. Ahtiainen, Eija K. Laakkonen, Pirjo Käkelä, Maisa Parviainen, Heikki Pohjolainen, Jarno Aaltonen, Ninamaria Onni, Koskimaa Mikko, Teemu J. Murtola, Heini Huhtala, Heikki Seikkula

**Affiliations:** ^1^ Department of Surgery Wellbeing Services County of Central Finland Jyväskylä Finland; ^2^ Faculty of Sport and Health Sciences University of Jyväskylä Jyväskylä Finland; ^3^ Institute of Clinical Medicine University of Eastern Finland Kuopio Finland; ^4^ Department of Surgery Kuopio University Hospital Kuopio Finland; ^5^ School of Medicine University of Eastern Finland Kuopio Finland; ^6^ Faculty of Medicine and Health Technology Tampere University Tampere Finland; ^7^ Department of Urology TAYS Cancer Center Tampere Finland; ^8^ Faculty of Social Sciences Tampere University Tampere Finland

**Keywords:** mortality risk, prostate cancer, prostate cancer prognosis, prostate cancer survival, survival, testosterone

## Abstract

**Background and Objective:**

The exact relationship between testosterone levels at diagnosis and prostate cancer (PCa) prognosis remains inadequately explored. The objective was to determine whether serum testosterone levels at the time of PCa diagnosis are associated with overall survival.

**Patients and Methods:**

The study cohort involved 2544 PCa patients, divided into three groups; normal (>10.4 nmol/L), grey zone (8.0–10.4 nmol/L) and low (2.0–8.0 nmol/L) serum testosterone groups. Survival outcomes were analysed using Kaplan–Meier curves and Cox regression models.

**Results:**

The analysis revealed an increased risk of death among patients with low testosterone levels compared to those with normal levels in uni‐ (HR = 1.67, 95% CI: 1.37–2.05, *p* < 0.001) and multivariable‐adjusted (HR = 1.58, 95% CI: 1.24–1.98, *p* < 0.001) analysis. Sensitivity analysis on patients with normal glucose metabolism revealed similar results (HR = 1.93, CI: 1.48–2.51, *p* < 0.001), as well as after stratified with age below 70 years (HR = 1.55, 95% CI: 1.02–2.36, *p* < 0.001) and over 70 years (HR = 1.83, 95% CI: 1.46–2.28, *p* < 0.001.) There was no difference in survival between the grey zone compared to other testosterone groups. The retrospective design limits our ability to infer causality.

**Conclusion:**

Low testosterone at the time of PCa diagnosis is an independent predictor of overall survival. Findings highlight the potential of testosterone for prognostic evaluation in PCa.

## 1 INTRODUCTION

Prostate cancer (PCa) is one of the most prevalent (globally 1.3 million new cases in the year 2018) malignancies affecting men globally.^1^ It is an androgen‐dependent malignancy.^2^ The main hormone related to PCa growth is testosterone.^3^ However, its role in PCa growth is currently conflicting,^4^, ^5^, ^6^ as well as it is relationship with prognosis,^4^, ^5^, ^7^ and survival.^1^, ^5^, ^6^, ^7^, ^8^

Factors known to influence testosterone levels include obesity, diabetes and metabolic syndromes,^9^ all of which are prevalent among adults and PCa patients.^10^ Approximately 650 million adults are affected by obesity.^11^ Obesity and metabolic syndromes are known to decrease testosterone levels^9^ and have been linked to more aggressive PCa characteristics, poorer survival outcomes and diabetes mellitus.^10^

Most of the studies evaluating the association between serum testosterone levels and PCa survival or progression have focused on early,^4^, ^5^, ^6^, ^7^, ^8^, ^12^ advanced^7^, ^8^, ^12^ or metastatic PCa.^13^, ^14^, ^15^, ^16^ Only a very limited number of studies have evaluated the association of testosterone measured at the time of PCa diagnosis with PCa prognosis.^17^ The ones that have have shown that low testosterone levels might be associated with worse PCa prognosis.^17^ However, none of the studies have examined the relationship between testosterone at diagnosis and overall survival.

The primary objective of this retrospective cohort study is to determine whether serum testosterone levels at the time of PCa diagnosis are associated with overall survival.

## 2 MATERIAL (PATIENTS) AND METHODS

The retrospective cohort included 2544 PCa patients, whose serum testosterone levels were measured at the time of PCa diagnosis (1 month before or a maximum of 3 months after diagnosis) from 2009 to 2023. Serum testosterone samples were acquired during the morning according to the standard clinical protocol in Finland. The data were collected and extracted from the medical records of Wellbeing Services County of Central Finland, Hospital Nova of Central Finland.

The study excluded (*n* = 1612) the following patients: (1) if serum testosterone levels were measured more than 3 months after PCa diagnosis; (2) if serum testosterone levels were measured more than 1 month before PCa diagnosis; (3) if serum testosterone levels were measured after the start of PCa treatment (e.g., androgen deprivation therapy [ADT]); (4) if testosterone levels were below 2 nmol/L at the time of PCa diagnosis to ensure the patients had not started any PCa treatment that could influence their serum testosterone levels.

The cohort was stratified into three groups based on the measured serum testosterone: normal (>10.4 nmol/L), grey zone (8.0–10.4 nmol/L) and low (2.0–8.0 nmol/L) testosterone levels. These groups were decided based on the current clinical reference ranges for serum testosterone, according to which testosterone levels ranging from 8.0 to 10.4 nmol/L can be considered grey zone, while testosterone levels below 8.0 nmol/L are considered the cut‐off level for hypogonadism.^18^, ^19^

Testosterone levels were measured and analysed by the following devices: Advia Centaur, Chemiluminescent Acridinium Ester Immunoassay Technology (2009 to 2010); Modular EVO, electrochemiluminescence immunoassay (ECLIA) method (2010 to 2015); A liquid chromatography–tandem mass spectrometry (LC–MS/MS) method (2015 to 2021); Shimadzu LCMS‐8060 NX, LC–MS/MS (2021 to 2023).

The primary outcome of the study was an overall risk of death, defined as the time from the date of PCa diagnosis to the date of death from any cause. Survival time for patients still alive at the end of the study or lost to follow‐up was censored on 31.12.2023. The median follow‐up time was 5.7 years interquartile range (IQR 3.1–8.9).

The statistical analysis was conducted using SPSS software (version 28, IBM Corp). Descriptive statistics were used to summarize the baseline characteristics. Continuous variables were described using medians and IQRs, while categorical variables were presented as counts and percentages.

Kaplan–Meier curves were plotted to illustrate the unadjusted effect of different testosterone levels on survival. Log‐rank tests were used to compare survival curves among the three predefined testosterone groups (normal, grey zone and low). Uni‐ and multivariable Cox proportional hazard regression models were used to evaluate mortality risk. The multivariable models were adjusted for potential confounding variables. These variables were age at PCa diagnosis, Prostate Specific Antigen (PSA), International Society of Urological Pathology (ISUP), comorbidities and body mass index (BMI). In the Cox regression analysis, the ISUP score was treated as a categorized variable. Sensitivity analysis was performed for patients with normal glucose metabolism. Normal glucose metabolism was defined based on the diagnosis of the patients so that they did not have diabetes mellitus, metabolic syndrome or hypertension. In our study, metabolic syndrome refers to metabolic syndrome, which is a cluster of conditions that increase the risk of cardiovascular disease and other health issues. These conditions typically include abdominal obesity, dyslipidaemia (high triglycerides or low HDL cholesterol), hypertension and insulin resistance or elevated fasting blood glucose. Stratified analysis was used given the potential modifying effect of age (<70 years and >70 years) to explore whether the impact of testosterone levels on survival differs by age.

We encountered a proportion of missing data in the ISUP Scores and BMI. The patients with missing ISUP Scores were excluded from the multivariable analysis. Only (*n* = 58) BMI records were found in medical records from the time of PCa diagnosis. Therefore, we used imputation for the BMI. The BMI was used only for the explorative subgroup analysis shown in the Appendix, Tables S4–S5. Predictors used for the imputation model were diabetes mellitus and metabolic syndrome and BMI from other time points (before or after diagnosis). The BMI data used from other time points are shown in Table S3 in the Appendix. The valid data were used for BMI imputation model in each group. The imputation method was monotone and type linear regression. In the subgroup analysis, pooled results are displayed. The subgroup analyses presented in this report are based on the imputed dataset. This approach allows us to utilize the full dataset, providing a more comprehensive analysis of the subgroups. By using imputed data, we aim to minimize the bias and variance that might arise from missing data.

## 3 RESULTS

### 3.1 Baseline patient characteristics

In total number 2544 PCa patients were included in the analysis. The groupwise number of patients was 1938 (76.2%) in the normal, 345 (13.6%) in the grey zone and 261 (10.3%) in the low testosterone group, Table 1. The median age at diagnosis was consistent across all groups at 72 years, (IQR 66–79). The median testosterone level was 16.0 nmol/L (IQR 13.0–19.9) for the normal group, 9.4 nmol/L (IQR 8.7–10.0) for the grey zone group and 6.5 nmol/L (IQR 5.2–7.4) for the low group. PSA levels at diagnosis varied, with the low testosterone group showing the highest median PSA at 5.2 ng/mL (IQR 2.7–19.5). The distribution of ISUP scores was consistent with a median score of 2 across all groups. However, higher grades (4–5) were more prevalent in the low testosterone group. There was a higher prevalence of diabetes in the grey zone (25.2%) and low (34.5%) testosterone groups compared to the normal group (15.6%), as well as metabolic syndrome. The initial treatments of PCa in different testosterone groups are shown in Appendix, Table S1–S2.

**TABLE 1:** Descriptive statistics of PCa patients in testosterone groups.

Characteristics	Testosterone >10.4 nmol/L	Testosterone 8.0–10.4 nmol/L	Testosterone 2.0–8.0 nmol/L
	*n* = 1938	*n* = 345	*n* = 261
Age at PCa diagnosis in years, median (IQR)	72 (66–78)	72 (65–78)	72 (65–79)
Testosterone in nmol/L, median (IQR)	16.0 (13.0–20.0)	9.4 (8.7–10.0)	6.5 (5.0–7.4)
PSA in ng/mL, median (IQR)	3.8 (2.3–10.7)	4.3 (2.3–12.7)	5.2 (2.7–19.5)
ISUP score, median (IQR)	2 (1–3)	2 (1–3)	2 (1–4)
ISUP score, *n* (%)			
1	562 (35.1)	101 (34.4)	61 (29.3)
2	285 (17.8)	48 (16.3)	32 (15.4)
3	294 (18.4)	48 (16.3)	36 (17.3)
4	228 (14.2)	64 (21.8)	37 (17.8)
5	62 (3.9)	8 (2.7)	20 (9.6)
Missing	171 (10.7)	25 (8.5)	22 (10.6)
Diabetes mellitus, *n* (%)	303 (15.6)	87 (25.2)	90 (34.5)
Metabolic syndrome, *n* (%)	187 (9.6)	57 (16.5)	66 (25.3)
Essential primary hypertension, *n* (%)	903 (46.6)	175 (50.7)	129 (49.4)
Myocardial infarction, *n* (%)	138 (7.1)	30 (8.7)	20 (7.7)
Atherosclerosis of aorta, *n* (%)	107 (5.5)	14 (4.1)	20 (7.7)
^a^BMI, median (IQR)	27.0 (24.0–31.0)	28.0 (26.5–33.0)	26.0 (21.0–37.0)
^b^BMI, median (IQR)	28.0 (24.0–34.0)	29.0 (25.0–35.0)	31.0 (25.0–37.0)

Abbreviation: IQR, interquartile range.

^a^ BMI are the original BMI values (*n* = 58).

^b^ BMI are the imputed pooled BMI values.

### 3.2 Survival analysis outcomes

**FIGURE 1:**
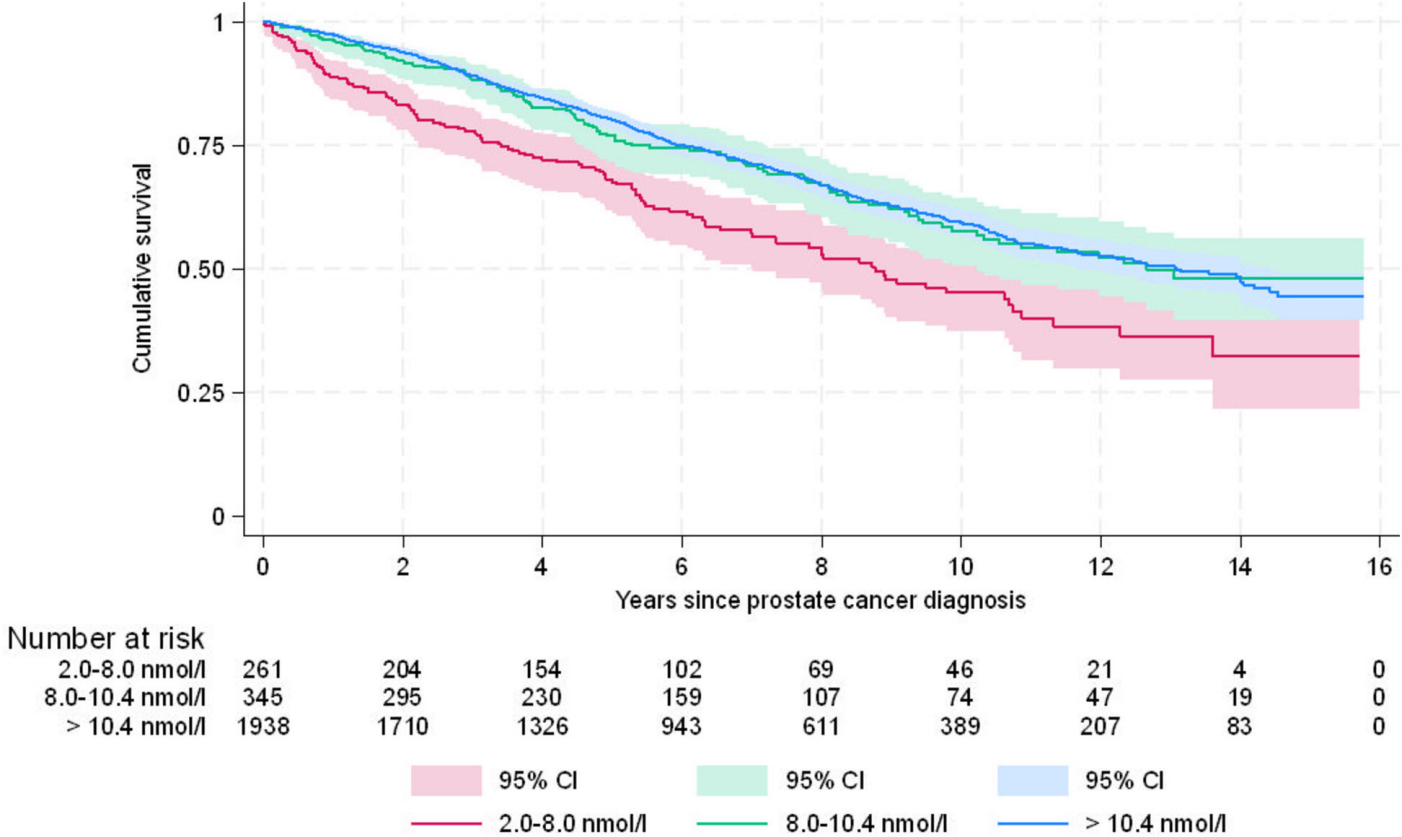
Cumulative overall survival in years after PCa diagnosis by testosterone level groups (*p* < 0.001 for difference by testosterone level).

The total number of deaths was 840 (33.0%) during the maximum follow‐up of 14‐years. Kaplan–Meier analysis revealed a significant (*p* < 0.001) difference in overall survival between testosterone groups. However, the pairwise comparison revealed a significant (*p* < 0.001) difference in survival only for the low testosterone (2.0–8.0 nmol/L) group compared to the normal (>10.4 nmol/L group), and also compared to the grey zone group (8.0–10.4 nmol/L) (both *p* < 0.001). Figure 1 presents the cumulative survival in different testosterone groups.

The uni‐ and multivariable Cox regression models, as summarized in Table 2, evaluated the mortality risk. The univariate model C‐index score was 0.53, and the multivariable was 0.72. ROC curves are shown in Figures S1–S2 in the Appendix. In univariable analyses, testosterone levels significantly predicted overall survival. Patients with low testosterone levels (2.0–8.0 nmol/L) exhibited a significantly higher risk of death (HR = 1.67, 95% CI: 1.37–2.04, *p* < 0.001) compared to those with normal testosterone levels (>10.4 nmol/L). The grey zone group (8.0–10.4 nmol/L) did not show a difference in risk of mortality HR = 1.03, (0.84–1.27), *p* = 0.616 compared to the normal testosterone group. After adjusting for multiple covariates, the risk of mortality remained increased for the low testosterone group HR = 1.58, (1.24–1.98), *p* < 0.001.

**TABLE 2:** Uni‐ and multivariable Cox regression models predicting risk of death.

Predictors	Univariable		Multivariable	
	HR (95% CI)	*p*‐Value	HR (95% CI)	*p*‐Value
Testosterone level (nmol/L)				
above 10.4	1		1	
8.0–10.4	1.03 (0.84–1.27)	0.616	1.04 (0.83–1.29)	0.759
2.0–8.0	1.67 (1.37–2.04)	<0.001	1.58 (1.24–1.98)	<0.001
PSA	1.05 (1.04–1.06)	<0.001	1.06 (1.04–1.07)	<0.001
ISUP Scores				
1	1		1	
2	1.29 (1.02–1.62)	0.034	1.27 (1.04–1.60)	0.046
3	1.93 (1.56–2.38)	<0.001	1.92 (1.55–2.36)	<0.001
4	2.83 (2.30–3.49)	<0.001	2.77 (2.24–3.42)	<0.001
5	4.47 (3.32–6.01)	<0.001	4.40 (3.27–5.94)	<0.001
Age at PCa diagnosis	1.11 (1.10–1.12)	<0.001	1.10 (1.09–1.11)	<0.001
Diabetes mellitus	1.02 (0.86–1.21)	0.825	0.98 (0.74–1.30)	0.884
Metabolic syndrome	0.88 (0.71–1.09)	0.228	0.76 (0.54–1.13)	0.196
Essential primary hypertension	0.76 (0.67–0.87)	<0.001	0.74 (0.63–0.88)	<0.001
Myocardial infarction	1.48 (1.19–1.84)	<0.001	1.32 (1.03–1.69)	0.031
Atherosclerosis of aorta	1.65 (1.30–2.10)	<0.001	1.50 (1.12–2.02)	0.007
^a^Sensitivity analysis				
Testosterone level (nmol/L)				
above 10.4	1		1	
8.0–10.4	1.06 (0.84–1.34)	0.609	1.0 (0.77–1.29)	0.993
2.0–8.0	2.03 (1.62–2.55)	<0.001	1.93 (1.48–2.51)	<0.001
PSA	1.05 (1.04–1.06)	<0.001	1.04 (1.03–1.06)	<0.001
ISUP Scores				
1	1		1	
2	1.32 (1.02–1.70)	0.037	1.30 (1.00–1.67)	0.048
3	2.04 (1.61–2.57)	<0.001	1.98 (1.57–2.51)	<0.001
4	3.07 (2.43–3.87)	<0.001	2.95 (2.33–3.74)	<0.001
5	4.57 (3.31–6.37)	<0.001	4.21 (3.02–5.87)	<0.001
Age at PCa diagnosis	1.10 (1.09–1.11)	<0.001	1.08 (1.06–1.09)	<0.001
Essential primary hypertension	0.75 (0.64–0.88)	<0.001	0.72 (0.60–0.86)	<0.001
Myocardial infarction	1.30 (0.99–1.71)	0.060	1.25 (0.93–1.70)	0.143
Atherosclerosis of aorta	1.51 (1.08–2.11)	0.017	1.55 (1.05–2.27)	0.026

*Note*: The multivariable model was adjusted for PSA, ISUP scores, age at PCa diagnosis, and comorbidities.

^a^ Sensitivity analysis on patients with normal glucose metabolism (*n* = 2064).

The increase in the risk of mortality in the low (2.0–8.0 nmol/L) testosterone group remained significant even after multivariable sensitivity HR = 1.93, (1.48–2.51), *p* < 0.001 and subgroup HR = 1.61, (1.45–1.79), *p* < 0.001 analysis. Sensitivity analysis was conducted on patients with normal glucose metabolism, while subgroup analysis included the imputed BMI values, Tables S3–S4.

**FIGURE 2:**
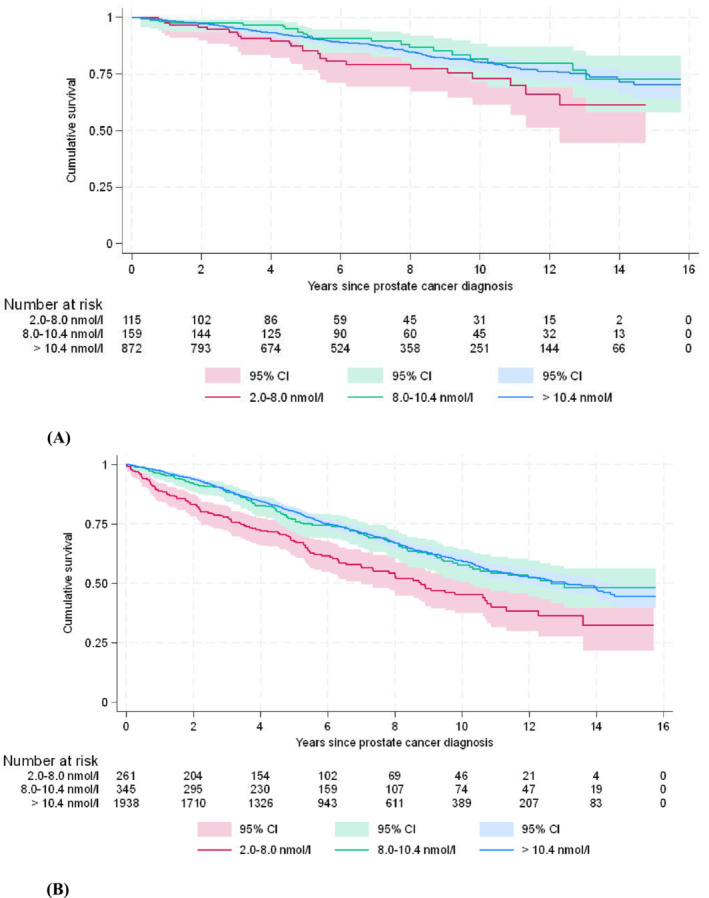
Stratified cumulative survival in years after PCa diagnosis in different testosterone level groups stratified by age group. (A) Males >70 years at PCa diagnosis (*p* < 0.001). (B) Males <70 years at PCa diagnosis (*p* < 0.001).

The stratified survival analysis by age revealed similar results as the primary survival analysis, indicating a significant (*p* < 0.001) difference in overall survival between low (8.0–2.0 nmol/L) testosterone group, normal (>10.4 nmol/L) and grey zone (8.0–10.4 nmol/L) groups. Kaplan–Meier curves for those above and under 70 years of old are shown in Figure 2.

The stratified by age Cox regression model showcased similarly increased risk of mortality only in low (2.0–8.0 nmol/L) testosterone group both among men under 70 years (univariable: HR = 1.55, 95% CI: 1.02–2.36, *p* = 0.032; multivariable: HR = 1.74, 95% CI: 1.09–2.80, *p* = 0.021) and above 70 years univariable: HR = 1.83, (1.46–2.28), *p* < 0.001; multivariable: HR = 1.67, (1.26–2.20), *p* < 0.001 old group, Table 3. There was no significant interaction between testosterone by age‐group (grey zone group*age‐group, HR = 1.28, 95% CI: 0.77–2.13, *p* = 0.350; low group*age‐group, HR = 1.17, 95% CI: 0.73–1.89, *p* = 0.507). Sensitivity multivariable analysis revealed only a significant increase in the risk of mortality above 70 years HR = 2.07, (1.52–2.80), *p* < 0.001 old, but not under 70 years HR = 1.73, (1.00–3.02), *p* = 0.052 old. Similarly, univariable subgroup analysis with imputed BMI values revealed only a significant increase in the risk of death only in those above 70 years HR = 1.74, (1.36–2.22), *p* < 0.001, but not in those under 70 years HR = 1.48, (0.95–2.31), *p* = 0.085 old. In contrast, the multivariable subgroup revealed a significant increase in the risk of mortality in both those under 70 years HR = 1.60, (1.01–2.53), *p* = 0.047 and above 70 years HR = 1.45, (1.12–1.88), *p* = 0.004 old. As well as subgroup sensitivity analysis for under 70 years, univariable: HR = 1.71, (1.01–2.90), *p* = 0.045; multivariable: HR = 1.76, (1.01–3.07), *p* = 0.047 and above 70 years, univariable: HR = 2.30, (1.73–3.06), *p* < 0.001; multivariable: HR = 2.07, (1.52–2.80), *p* < 0.001. Subgroup and subgroup sensitivity analysis results can be found in the Supporting information.

**TABLE 3:** Uni‐ and multivariable Cox regression models predicting risk of death stratified by age at PCa diagnosis.

Predictors	Univariable		Multivariable	
	HR (95% CI)	*p*‐Value	HR (95% CI)	*p*‐Value
**Age <70 at PCa diagnosis**				
Testosterone level (nmol/L)				
above 10.4	1		1	
8.0–10.4	0.86 (0.54–1.36)	0.611	0.62 (0.36–1.08)	0.089
2.0–8.0	1.55 (1.02–2.36)	0.032	1.74 (1.09–2.80)	0.021
PSA	1.02 (1.01–1.03)	<0.001	1.03 (1.01–1.05)	0.014
ISUP Scores				
1	1		1	
2	1.69 (1.06–2.71)	0.029	1.74 (1.02–2.95)	0.041
3	2.60 (1.67–4.05)	<0.001	2.89 (1.78–4.70)	<0.001
4	5.17 (3.33–8.02)	<0.001	5.88 (3.71–9.32)	<0.001
5	2.60 (1.03–6.54)	0.043	2.79 (1.10–7.10)	0.031
Diabetes mellitus	1.8 (0.89–1.33)	0.440	1.07 (0.58–1.97)	0.822
Metabolic syndrome	0.58 (0.33–1.02)	0.059	0.48 (0.20–1.11)	0.100
Essential primary hypertension	0.77 (0.56–1.05)	0.100	0.74 (0.51–1.06)	0.264
Myocardial infarction	1.26 (0.70–2.26)	0.448	1.45 (0.75–2.79)	0.264
Atherosclerosis of aorta	1.67 (0.88–3.17)	0.116	1.55 (0.74–3.25)	0.244
^a^Sensitivity analysis				
Testosterone level (nmol/L)				
above 10.4	1		1	
8.0–10.4	0.89 (0.53–1.51)	0.673	0.70 (0.39–1.25)	0.227
2.0–8.0	1.71 (1.01–2.90)	0.045	1.73 (1.00–3.02)	0.052
PSA	1.04 (1.02–1.07)	<0.001	1.03 (1.00–1.05)	0.030
ISUP Scores				
1	1		1	
2	1.86 (1.12–3.09)	0.017	1.77 (1.07–2.95)	0.027
3	2.82 (1.73–4.62)	<0.001	2.70 (1.64–4.43)	<0.001
4	5.57 (3.41–9.09)	<0.001	5.67 (3.40–9.45)	<0.001
5	3.57 (1.40–9.10)	0.008	3.44 (1.33–8.89)	<0.001
Essential primary hypertension	0.82 (0.58–1.17)	0.275	0.73 (0.49–1.07)	0.104
Myocardial infarction	1.05 (0.52–2.15)	0.891	1.02 (0.44–2.35)	0.962
Atherosclerosis of aorta	1.84 (0.81–4.16)	0.146	1.33 (0.47–3.74)	0.591
**Age >70 at PCa diagnosis**				
Testosterone level (nmol/L)				
above 10.4	1		1	
8.0–10.4	1.09 (0.87–1.37)	0.451	1.10 (0.84–1.45)	0.413
2.0–8.0	1.83 (1.46–2.28)	<0.001	1.67 (1.26–2.20)	<0.001
PSA	1.06 (1.03–1.09)	<0.001	1.09 (1.07–1.12)	<0.001
ISUP Scores				
1	1		1	
2	1.03 (0.79–1.35)	0.823	1.02 (0.78–1.33)	0.880
3	1.42 (1.12–1.80)	0.004	1.40 (1.10–1.78)	0.006
4	1.76 (1.39–2.23)	<0.001	1.63 (1.26–2.11)	<0.001
5	3.76 (2.73–5.18)	<0.001	3.37 (2.44–4.66)	<0.001
Diabetes	1.00 (0.91–1.11)	0.974	1.08 (0.78–1.48)	0.642
Metabolic syndrome	0.91 (0.72–1.17)	0.468	0.91 (0.60–1.38)	0.643
Essential primary hypertension	0.70 (0.60–0.82)	<0.001	0.75 (0.62–0.90)	0.002
Myocardial infarction	1.13 (0.88–1.45)	0.336	1.12 (0.85–1.48)	0.404
Atherosclerosis of aorta	1.23 (0.93–1.62)	0.146	1.18 (0.85–1.63)	0.318
^a^Sensitivity analysis				
Testosterone level (nmol/L)				
above 10.4	1		1	
8.0–10.4	1.11 (0.84–1.46)	0.464	1.12 (0.84–1.49)	0.426
2.0–8.0	2.30 (1.73–3.06)	<0.001	2.07 (1.52–2.80)	<0.001
PSA	1.08 (1.06–1.10)	<0.001	1.09 (1.06–1.11)	<0.001
ISUP Scores				
1	1		1	
2	1.03 (0.76–1.38)	0.868	1.03 (0.76–1.39)	0.867
3	1.46 (1.12–1.90)	0.005	1.43 (1.10–1.87)	0.008
4	1.91 (1.47–2.48)	<0.001	1.78 (1.36–2.33)	<0.001
5	3.57 (2.50–5.09)	<0.001	3.01 (2.09–4.32)	<0.001
Essential primary hypertension	0.67 (0.55–0.80)	<0.001	0.73 (0.60–0.89)	0.002
Myocardial infarction	1.17 (0.87–1.57)	0.300	1.18 (0.85–1.63)	0.328
Atherosclerosis of aorta	1.18 (0.81–1.71)	0.385	1.22 (0.80–1.85)	0.358

*Note*: The multivariable model was adjusted for PSA, ISUP scores, age at PCa diagnosis, and comorbidities.

^a^ Sensitivity analysis on patients with normal glucose metabolism (*n* = 2064).

## 4 DISCUSSION

Our study establishes significant associations between serum testosterone levels at the time of PCa diagnosis and overall survival. We have demonstrated that low testosterone levels (2.0–8.0 nmol/L) are linked to shorter overall survival and a higher risk of mortality, reinforcing the potential role of testosterone as a prognostic biomarker in PCa. This relationship persisted even in patients with normal glucose metabolism and was a bit higher in patients over 70 years old. Our analysis did not find significant interaction effects between testosterone levels and age group, suggesting that the impact of testosterone levels on PCa survival is consistent across different age groups.

The finding that low serum testosterone levels at diagnosis are associated with poorer survival aligns with prior studies, such as those by García‐Cruz et al.,^17^ which found that lower testosterone levels increase the progression of PCa. Our study extends this knowledge by providing robust evidence that low testosterone levels are an independent predictor of mortality.

These results challenge the traditional understanding of PCa as an androgen‐dependent disease, which suggests that higher testosterone levels might contribute to disease progression.^20^ Recent evidence, including our findings, supports a paradigm shift, indicating that higher testosterone levels do not increase the risk of PCa,^21^, ^22^ nor does it lead to worse outcomes or a higher rate of recurrence.^23^ In addition, neither do low serum testosterone levels protect against PCa.^23^ This is consistent with the saturation model, where PCa progression reaches a plateau at relatively low testosterone concentrations and further increases in testosterone do not exacerbate disease progression.^20^

Furthermore, recent meta‐analyses have highlighted the complex role of testosterone in PCa prognosis, suggesting that lower testosterone levels are linked with decreased mortality and slower disease progression during ADT. Conversely, higher testosterone levels appear to be protective before ADT and in advanced PCa.^24^

Currently, there are only a limited number of studies evaluating the association of serum testosterone levels at PCa diagnosis and PCa prognosis.^24^ However, there are few studies that have evaluated early PCa serum testosterone levels association with PCa prognosis and progression. Most of the previous studies have demonstrated that serum testosterone levels below 10.4 nmol/L do not lead to an increased risk of biochemical recurrence^25^ or radiotherapy or ADT outcomes.^26^ However, some are indicating contradictory results, showcasing that testosterone levels below 10.4 nmol/L are associated with PSA failure‐free survival^27^ and a higher risk of biochemical failure.^28^ In our study, we did not find a difference in survival between groups that had testosterone levels 8.0–10.4 nmol/L and above 10.4 nmol/L.

A limited number of studies have used cut‐off serum testosterone levels below 8.0 nmol/L as we did. In these studies, below 8.0 nmol/L serum testosterone levels were associated with a predominance of Gleason pattern 4–5.^25^ This finding complemented by our results could indicate that lower serum testosterone levels at the PCa diagnosis or during the early phase could be linked to a higher risk of more aggressive form of PCa or castration‐resistant PCa. This hypothesis is supported by the recent meta‐analysis.^24^

It could also be that low testosterone (2.0–8.0 nmol/L) could be linked to a more aggressive form of PCa. This was evident in our cohort, higher prevalence of ISUP scores of 4 and 5 in the low testosterone group. Similar findings have been observed in previous studies.^25^ While higher ISUP grade has been associated with increased PCa‐specific risk of mortality.^29^

On the other hand, males with low testosterone usually have a higher prevalence of comorbidities, besides PCa,^9^, ^10^ reflecting a weaker overall state of health. In our study, we tried to evaluate this and minimize the effect of the most prevalent comorbidities, by incorporating prevalent comorbidities associated with low testosterone, such as metabolic syndrome, non‐normal glucose metabolism and obesity in our statistical models.

Our study's retrospective design limits the ability to infer causality definitively. In addition, as we did look at the specific cause of death in our cohort. We cannot be certain, which cause of death was increased in the 2.0–8.0 nmol/L testosterone group. Our study was missing other unmeasured variables, including detailed TNM staging, which could influence the observed associations. The use of multiple imputations for missing BMI data, while methodologically sound, introduces some level of uncertainty. Future prospective studies should aim to validate our findings in different PCa stages and explore the underlying mechanisms linking low testosterone levels to poorer PCa outcomes.

Clinicians should consider incorporating testosterone measurements into routine diagnostic protocols for PCa to better stratify patients' risk and tailor treatment plans accordingly. This approach could help identify high‐risk patients who might benefit from more aggressive or alternative therapeutic interventions.

## 5 CONCLUSIONS

In conclusion, our study highlights the prognostic value of low serum testosterone levels at PCa diagnosis. These findings underscore the need for a nuanced understanding of the role of androgens in PCa and support the integration of testosterone level assessment into clinical practice to improve patient outcomes. We suggest that the evaluation of the individual testosterone level of the patient will help clinicians predict the patient's prognosis and help to identify patients who might benefit from more aggressive therapeutic approaches.

## AUTHOR CONTRIBUTIONS

All authors have made significant contributions to this research work. Ilkka Jussila analyzed the data, drafted and revised the manuscript, as well as overseeing the whole research project. Maisa Parviainen, Heikki Pohjola, Jarno Aaltonen, Ninamaria Onni and Mikko Koskimaa were involved in the data collection and editing of the manuscript. Juha P. Ahtiainen, Eija K. Laakkonen, Pirjo Käkelä and Teemu J. Murtola provided critical feedback, edited the manuscript and assisted with the interpretation of the results. Heini Huhtala provided critical data analysis feedback, edited the manuscript and assisted interpretation of the results. Heikki Seikkula was the study sponsor responsible for conceptualization, and design of the study, as well as validating the findings, and editing the manuscript.

## CONFLICT OF INTEREST STATEMENT

TJ Murtola: Lecture fees from Astellas, Amgen, Janssen, Novartis, and Sanofi, paid consultant for Astellas, Janssen Pfizer, and Accord, clinical trial funding from Bayer, Pfizer, and Janssen.

## Supporting information


**Table S1.** Initial treatments of PCa in different testosterone groups.
**Table S2.** Initial treatments of PCa stratified by age in different testosterone groups.
**Table S3.** BMI data used for BMI imputation.
**Table S4**. Uni‐ and multivariable Cox regression models of BMI subgroup analysis.
**Table S5**. Uni‐ and multivariable Cox regression models of BMI subgroup analysis stratified by age.
**Figure S1.** ROC Curve of univariable Cox regression model between testosterone groups and risk of mortality.
**Figure S2.** ROC Curve of multivariable Cox regression model between testosterone groups and risk of mortality. The multivariable model was adjusted for PSA, ISUP scores, age at PCa diagnosis, and comorbidities.
