# Supplementary material for: Testosterone levels at diagnosis: A key predictor of overall survival among patients with prostate cancer
Source: BJUI Compass. 2025 Feb 17;6(2):e484. doi: 10.1002/bco2.484 (PMC11832296; doi:10.1002/bco2.484)
Supplement: Supplementary file 1 — Table S1. Initial treatments of PCa in different testosterone groups. Table S2. Initial treatments of PCa stratified by age in different testosterone groups. Table S3. BMI data used for BMI imputation. Table S4. Uni‐ and multivariable Cox regression models of BMI subgroup analysis. Table S5. Uni‐ and multivariable Cox regression models of BMI subgroup analysis stratified by age. Figure S1. ROC Curve of univariable Cox regression model between testosterone groups and risk of mortality. Figure S2. ROC Curve of multivariable Cox regression model between testosterone groups and risk of mortality. The multivariable model was adjusted for PSA, ISUP scores, age at PCa diagnosis, and comorbidities. [file BCO2-6-e484-s001.docx]

**Appendix A. Supplementary data**

**Supplementary Table I.** Initial treatments of PCa in different testosterone groups.

| **Initial treatment of PCa** | **Testosterone**  **>10.4 nmol/l**  **n=1938** | **Testosterone**  **8.0-10.4 nmol/l**  **n=345** | **Testosterone**  **2.0-8.0 nmol/l**  **n= 261** |
| --- | --- | --- | --- |
| ^i^ Radical prostatectomy |  |  |  |
| ^i^ Laparoscopic radical prostatectomy, n (%) | 197 (11.6) | 32 (9.3) | 19 (7.3) |
| ^i^ Robot-assisted laparoscopic prostatectomy, n (%) | 68 (3.5) | 13 (3.8) | 7 (2.7) |
| ^ii^ Radiation therapy as monotherapy |  |  |  |
| ^ii^ Radical radiation therapy, n (%) | 273 (14.1) | 48 (13.9) | 24 (9.2) |
| ^ii^ Palliative radiation therapy, n (%) | 22 (1.1) | 4 (1.2) | 5 (1.9) |
| Androgen deprivation therapy |  |  |  |
| Monotherapy, n (%) | 659 (34.0) | 127 (36.8) | 119 (45.6) |
| ^ii^ In combination with radiation therapy, n (%) | 124 (6.4) | 20 (5.8) | 17 (6.5) |
| Active/passive surveillance, n (%) | 595 (29.7) | 101 (29.3) | 72 (27.6) |

^i^ Data about radiation therapy was only found from the year 2014 onwards.

^ii^ Data about radical prostatectomy or robot-assisted laparoscopic prostatectomy was only available from 2017 onwards. Radical prostatectomy surgeries before the year 2017 were conducted in another hospital.

**Supplementary Table II.** Initial treatments of PCa stratified by age in different testosterone groups.

| **Initial treatment of PCa stratified by age** | **Testosterone**  **>10.4 nmol/l**  **n=1938** | **Testosterone**  **8.0-10.4 nmol/l**  **n=345** | **Testosterone**  **2.0-8.0 nmol/l**  **n= 261** |
| --- | --- | --- | --- |
| **^a^ Age <= 70 years at PCa diagnosis** |  |  |  |
| ^i^ Radical prostatectomy |  |  |  |
| ^i^ Laparoscopic radical prostatectomy, n (%) | 169 (19.3) | 28 (17.6) | 19 (16.5) |
| ^i^ Robot-assisted laparoscopic prostatectomy, n (%) | 61 (7.0) | 13 (8.2) | 7 (6.1) |
| ^ii^ Radiation therapy as monotherapy |  |  |  |
| ^ii^ Radical radiation therapy, n (%) | 119 (13.6) | 23 (14.5) | 15 (13.0) |
| ^ii^ Palliative radiation therapy, n (%) | 12 (1.4) | 1 (0.6) | 3 (2.6) |
| Androgen deprivation therapy |  |  |  |
| Monotherapy, n (%) | 186 (21.3) | 39 (24.5) | 32 (27.8) |
| ^ii^ In combination with radiation therapy, n (%) | 63 (7.2) | 8 (5.0) | 9 (7.8) |
| Active/passive surveillance, n (%) | 264 (30.2) | 47 (29.6) | 30 (26.1) |
| **^b^ Age 71 – 80 years at PCa diagnosis** |  |  |  |
| ^i^ Radical prostatectomy |  |  |  |
| ^i^ Laparoscopic radical prostatectomy, n (%) | 27 (3.5) | 4 (3.1) | 0 (0.0) |
| ^i^ Robot-assisted laparoscopic prostatectomy, n (%) | 7 (0.9) | 0 (0.0) | 0 (0.0) |
| ^ii^ Radiation therapy as monotherapy |  |  |  |
| ^ii^ Radical radiation therapy, n (%) | 137 (17.7) | 22 (17.2) | 8 (8.3) |
| ^ii^ Palliative radiation therapy, n (%) | 8 (1.0) | 1 (0.8) | 1 (1.0) |
| Androgen deprivation therapy |  |  |  |
| Monotherapy, n (%) | 316 (40.9) | 57 (44.5) | 60 (62.5) |
| ^ii^ In combination with radiation therapy, n (%) | 56 (7.2) | 9 (7.0) | 8 (8.3) |
| Active/passive surveillance, n (%) | 222 (28.7) | 35 (27.3) | 19 (19.8) |
| **^c^ Age >= 81 years at PCa diagnosis** |  |  |  |
| ^i^ Radical prostatectomy |  |  |  |
| ^i^ Laparoscopic radical prostatectomy, n (%) | 1 (0.3) | 0 (0.0) | 0 (0.0) |
| ^i^ Robot-assisted laparoscopic prostatectomy, n (%) | 0 (0.0) | 0 (0.0) | 0 (0.0) |
| ^ii^ Radiation therapy as monotherapy |  |  |  |
| ^ii^ Radical radiation therapy, n (%) | 17 (5.8) | 3 (5.1) | 1 (2.0) |
| ^ii^ Palliative radiation therapy, n (%) | 2 (0.7) | 2 (3.4) | 1 (2.0) |
| Androgen deprivation therapy |  |  |  |
| Monotherapy, n (%) | 157 (53.4) | 31 (52.5) | 27 (52.9) |
| ^ii^ In combination with radiation therapy, n (%) | 5 (1.7) | 3 (5.1) | 0 (0.0) |
| Active/passive surveillance, n (%) | 112 (38.1) | 20 (33.9) | 22 (43.1) |

^i^ Data about radiation therapy was only found from the year 2014 onwards.

^ii^ Data about radical prostatectomy or robot-assisted laparoscopic prostatectomy was only available from 2017 onwards. Radical prostatectomy surgeries before the year 2017 were conducted in another hospital.

^a^ Age <= 70 years at PCa diagnosis, included (n = 115) patients in the 2.0 – 8.0 nmol/l testosterone group, (n = 159) in the 8.0 – 10.4 nmol/l testosterone group, and (n = 874) in the > 10.4 nmol/l testosterone group.
^b^ Age 71 - 80 years at PCa diagnosis, included (n = 96) patients in the 2.0 – 8.0 nmol/l testosterone group, (n = 128) in the 8.0 – 10.4 nmol/l testosterone group, and (n = 773) in the > 10.4 nmol/l testosterone group.
^c^ Age >= 81 years at PCa diagnosis, included (n = 51) patients in the 2.0 – 8.0 nmol/l testosterone group, (n = 59) in the 8.0 – 10.4 nmol/l testosterone group, and (n = 294) in the > 10.4 nmol/l testosterone group.

**Supplementary Table III.** BMI data used for BMI imputation.

| **BMI** | **Testosterone**  **>10.4 nmol/l**  **n=1938** | **Testosterone**  **8.0-10.4 nmol/l**  **n=345** | **Testosterone**  **2.0-8.0 nmol/l**  **n= 261** |
| --- | --- | --- | --- |
| Valid | 1009 | 189 | 146 |
| Missing | 929 | 156 | 115 |
| Median (IQR) | 26.0 (24.0 – 30.0) | 28.0 (26.0 – 31.0) | 31.00 (27.0 – 35.0) |

**Supplementary Table IV**. Uni- and multivariable Cox regression models of BMI subgroup analysis.

| **Predictors** | **Univariable** |  |  | **Multivariable** |  |
| --- | --- | --- | --- | --- | --- |
|  | **HR (95% CI)** | **P-value** |  | **HR (95% CI)** | **P-value** |
| Subgroup analysis |  |  |  |  |  |
| Testosterone level (nmol/l) |  |  |  |  |  |
| above 10.4 | 1 |  |  | 1 |  |
| 8.0 - 10.4 | 1.01 (0.93 – 1.10) | 0.800 |  | 1.02 (0.92 – 1.12) | 0.776 |
| 2.0 – 8.0 | 1.55 (1.42 – 1.69) | <0.001 |  | 1.61 (1.45 – 1.79) | <0.001 |
| PSA | 1.05 (1.05 – 1.05) | <0.001 |  | 1.06 (1.05 – 1.06) | <0.001 |
| ISUP Scores |  |  |  |  |  |
| 1 | 1 |  |  | 1 |  |
| 2 | 1.29 (1.17 – 1.41) | <0.001 |  | 1.05 (0.94 – 1.16) | 0.401 |
| 3 | 1.93 (1.77 – 2.10) | <0.001 |  | 1.47 (1.33 – 1.61) | <0.001 |
| 4 | 2.83 (2.60 – 3.08) | <0.001 |  | 2.0 (1.82 – 2.20) | <0.001 |
| 5 | 4.47 (3.96 – 5.04) | <0.001 |  | 2.80 (2.44 – 3.21) | <.001 |
| Age at PCa diagnosis | 1.10 (1.10 – 1.11) | <0.001 |  | 1.10 (1.09 – 1.10) | <0.001 |
| Diabetes Mellitus | 0.97 (0.90 – 1.04) | 0.351 |  | 0.97 (0.85 – 1.10) | 0.596 |
| Metabolic syndrome | 0.84 (0.77 – 0.92) | <0.001 |  | 0.94 (0.80 – 1.10) | 0.434 |
| Essential primary hypertension | 0.76 (0.72 – 0.81) | <0.001 |  | 0.70 (0.65 – 0.75) | <0.001 |
| Myocardial infarction | 1.33 (1.21 – 1.46) | <0.001 |  | 1.06 (0.95 – 1.19) | 0.318 |
| Atherosclerosis of aorta | 1.53 (1.38 – 1.70) | <0.001 |  | 1.19 (1.04 – 1.36) | 0.010 |
| BMI | 1.00 (1.00 – 1.00) | 0.032 |  | 1.00 (0.99 – 1.01) | 0.851 |
| ^a^Sensitivity analysis |  |  |  |  |  |
| Testosterone level (nmol/l) |  |  |  |  |  |
| above 10.4 | 1 |  |  | 1 |  |
| 8.0 - 10.4 | 1.08 (0.88 – 1.32) | 0.455 |  | 0.99 (0.80 – 1.22) | 0.891 |
| 2.0 – 8.0 | 1.98 (1.63 – 2.40) | <0.001 |  | 1.98 (1.60 – 2.45) | <0.001 |
| PSA | 1.05 (1.04 – 1.06) | <0.001 |  | 1.05 (1.04 – 1.06) | <0.001 |
| ISUP Scores |  |  |  |  |  |
| 1 | 1 |  |  | 1 |  |
| 2 | 1.28 (1.05 – 1.58) | 0.017 |  | 0.95 (0.77 – 1.17) | 0.623 |
| 3 | 2.14 (1.78 – 2.57) | <0.001 |  | 1.52 (1.26 – 1.83) | <0.001 |
| 4 | 2.93 (2.42 – 3.54) | <0.001 |  | 2.02 (1.66 – 2.46) | <0.001 |
| 5 | 5.22 (4.12 – 6.61) | <0.001 |  | 2.71 (2.10 – 3.59) | <0.001 |
| Age at PCa diagnosis | 1.10 (1.09 – 1.10) | <0.001 |  | 1.09 (1.08 – 1.10) | <0.001 |
| Essential primary hypertension | 0.71 (0.63 – 0.81) | <0.001 |  | 0.64 (0.55 – 0.73 | <0.001 |
| Myocardial infarction | 1.35 (1.10 – 1.65) | 0.004 |  | 0.99 (0.79 – 1.25) | 0.954 |
| Atherosclerosis of aorta | 1.46 (1.14 – 1.87) | 0.003 |  | 1.09 (0.83 – 1.44) | 0.549 |
| BMI | 1.00 (0.98 – 1.02) | 0.826 |  | 1.00 (0.98 – 1.02) | 0.984 |

Subgroup analysis with imputated BMI values, showcasing the pooled results (n=2544x5). ^a^Sensitivity analysis with imputed BMI values on patients with normal glucose metabolism, showcasing the pooled results (n=2544x5). The multivariable model was adjusted for PSA, ISUP scores, age at PCa diagnosis, BMI, and comorbidities.

**Supplementary Table V**. Uni- and multivariable Cox regression models of BMI subgroup analysis stratified by age.

|  |  | **Univariable** |  |  | **Multivariable** |  |
| --- | --- | --- | --- | --- | --- | --- |
| **Predictors** |  | **HR (95% CI)** | **P-value** |  | **HR (95% CI)** | **P-value** |
| **Age < 70 at PCa diagnosis** |  |  |  |  |  |  |
| Subgroup analysis |  |  |  |  |  |  |
| Testosterone level (nmol/l) |  |  |  |  |  |  |
| above 10.4 |  | 1 |  |  | 1 |  |
| 8.0 - 10.4 |  | 0.81 (0.49 – 1.32) | 0.394 |  | 0.68 (0.41 – 1.14) | 0.146 |
| 2.0 – 8.0 |  | 1.48 (0.95 – 2.31) | 0.085 |  | 1.60 (1.01 – 2.53) | 0.047 |
| PSA |  | 1.05 (1.03 – 1.07) | <0.001 |  | 1.03 (1.01 – 1.05) | 0.005 |
| ISUP Scores |  |  |  |  |  |  |
| 1 |  | 1 |  |  | 1 |  |
| 2 |  | 1.26 (0.66 – 2.40) | 0.479 |  | 0.79 (0.43 – 1.45) | 0.441 |
| 3 |  | 1.94 (1.04 – 3.60) | 0.037 |  | 1.29 (0.67 – 2.46) | 0.448 |
| 4 |  | 3.88 (2.09 – 7.20) | <0.001 |  | 2.00 (1.07 – 3.74) | 0.030 |
| 5 |  | 1.94 (0.70 – 5.38) | 0.205 |  | 4.65 (2.47 – 8.77) | <0.001 |
| Diabetes Mellitus |  | 0.76 (0.50 – 1.15) | 0.190 |  | 1.13 (0.56 – 2.27) | 0.773 |
| Metabolic syndrome |  | 0.58 (0.33 – 1.02) | 0.059 |  | 0.43 (0.18 – 1.03 | 0.059 |
| Essential primary hypertension |  | 0.77 (0.56 – 1.05) | 0.100 |  | 0.79 (0.56 – 1.11) | 0.172 |
| Myocardial infarction |  | 1.26 (0.70 – 2.26) | 0.448 |  | 1.59 (0.87 – 2.90 | 0.132 |
| Atherosclerosis of aorta |  | 1.67 (0.88 – 3.17) | 0.116 |  | 1.67 (0.85 – 3.27) | 0.134 |
| BMI |  | 0.99 (0.94 – 1.04) | 0.557 |  | 0.99 (0.94 – 1.05) | 0.800 |
| ^a^Sensitivity analysis |  |  |  |  |  |  |
| Testosterone level (nmol/l) |  |  |  |  |  |  |
| above 10.4 |  | 1 |  |  | 1 |  |
| 8.0 - 10.4 |  | 0.89 (0.53 – 1.51) | 0.673 |  | 0.71 (0.39 – 1.27) | 0.243 |
| 2.0 – 8.0 |  | 1.71 (1.01 – 2.90) | 0.045 |  | 1.76 (1.01 – 3.07) | 0.047 |
| PSA |  | 1.04 (1.02 – 1.07) | <0.001 |  | 1.03 (1.00 – 1.05) | 0.036 |
| ISUP Scores |  |  |  |  |  |  |
| 1 |  | 1 |  |  | 1 |  |
| 2 |  | 1.86 (1.12 – 3.09) | 0.017 |  | 1.81 (1.08 – 3.01) | 0.024 |
| 3 |  | 2.82 (1.73 – 4.62) | <0.001 |  | 2.70 (1.64 – 4.44) | <0.001 |
| 4 |  | 5.57 (3.41 – 9.09) | <0.001 |  | 5.63 (3.38 – 9.39) | <0.001 |
| 5 |  | 3.57 (1.40 – 9.10) | 0.008 |  | 3.34 (1.30 – 8.76) | 0.012 |
| Essential primary hypertension |  | 0.82 (0.58 – 1.17) | 0.275 |  | 0.73 (0.49 – 1.07) | 0.112 |
| Myocardial infarction |  | 1.05 (0.52 – 2.15) | 0.891 |  | 1.02 (0.44 – 2.34) | 0.973 |
| Atherosclerosis of aorta |  | 1.84 (0.81 – 4.16) | 0.146 |  | 1.32 (0.47 – 3.73) | 0.602 |
| BMI |  | 0.99 (0.94 – 1.05) | 0.695 |  | 0.99 (0.94 – 1.04) | 0.688 |
| **Age > 70 at PCa diagnosis** |  |  |  |  |  |  |
| Subgroup analysis |  |  |  |  |  |  |
| Testosterone level (nmol/l) |  |  |  |  |  |  |
| above 10.4 |  | 1 |  |  | 1 |  |
| 8.0 - 10.4 |  | 1.07 (0.85 – 1.35) | 0.564 |  | 1.08 (0.85 – 1.36) | 0.546 |
| 2.0 – 8.0 |  | 1.74 (1.36 – 2.22) | <0.001 |  | 1.45 (1.12 – 1.88) | 0.004 |
| PSA |  | 1.08 (1.06 – 1.10) | <0.001 |  | 1.07 (1.05 – 1.09) | <0.001 |
| ISUP Scores |  |  |  |  |  |  |
| 1 |  | 1 |  |  | 1 |  |
| 2 |  | 0.91 (0.65 – 1.26) | 0.563 |  | 0.97 (0.69 – 1.36) | 0.858 |
| 3 |  | 1.25 (0.92 – 1.71) | 0.151 |  | 1.34 (0.98 – 1.84) | 0.067 |
| 4 |  | 1.55 (1.14 – 2.11) | 0.005 |  | 1.59 (1.17 – 2.17) | 0.003 |
| 5 |  | 3.37 (2.32 – 4.90) | <0.001 |  | 3.39 (2.31 – 4.96) | <0.001 |
| Diabetes Mellitus |  | 1.02 (0.84 – 1.24) | 0.842 |  | 1.04 (0.74 – 1.47) | 0.835 |
| Metabolic syndrome |  | 0.91 (0.72 – 1.17) | 0.468 |  | 0.93 (0.62 – 1.39) | 0.723 |
| Essential primary hypertension |  | 0.70 (0.60 – 0.82) | <0.001 |  | 0.74 (0.62 – 0.88) | <0.001 |
| Myocardial infarction |  | 1.13 (0.88 – 1.45) | 0.336 |  | 1.13 (0.87 – 1.45) | 0.366 |
| Atherosclerosis of aorta |  | 1.23 (0.93 – 1.62) | 0.146 |  | 1.34 (1.01 – 1.79) | 0.043 |
| BMI |  | 1.00 (0.98 – 1.02) | 0.762 |  | 1.01 (0.99 – 1.02) | 0.560 |
| ^a^Sensitivity analysis |  |  |  |  |  |  |
| Testosterone level (nmol/l) |  |  |  |  |  |  |
| above 10.4 |  | 1 |  |  | 1 |  |
| 8.0 - 10.4 |  | 1.10 (0.84 – 1.46) | 0.464 |  | 1.13 (0.85 – 1.50) | 0.414 |
| 2.0 – 8.0 |  | 2.30 (1.73 – 3.06) | <0.001 |  | 2.07 (1.52 – 2.80 | <0.001 |
| PSA |  | 1.08 (1.06 – 1.10) | <0.001 |  | 1.09 (1.07 – 1.11) | <0.001 |
| ISUP Scores |  |  |  |  |  |  |
| 1 |  | 1 |  |  | 1 |  |
| 2 |  | 1.02 (0.76 – 1.39) | 0.868 |  | 1.03 (0.76 – 1.39) | 0.873 |
| 3 |  | 1.46 (1.12 – 1.90) | 0.005 |  | 1.43 (1.10 – 1.87) | 0.009 |
| 4 |  | 1.91 (1.47 – 2.48) | <0.001 |  | 1.78 (1.36 – 2.33) | <0.001 |
| 5 |  | 3.57 (2.50 – 5.09) | <0.001 |  | 3.01 (2.09 – 4.35) | <0.001 |
| Essential primary hypertension |  | 0.67 (0.55 – 0.80) | <0.001 |  | 0.73 (0.60 – 0.89) | 0.002 |
| Myocardial infarction |  | 1.17 (0.87 – 1.57) | 0.300 |  | 1.18 (0.85 – 1.63) | 0.330 |
| Atherosclerosis of aorta |  | 1.18 (0.81 – 1.71) | 0.385 |  | 1.21 (0.79 – 1.84) | 0.380 |
| BMI |  | 1.00 (0.98 – 1.02) | 0.892 |  | 1.00 (0.98 – 1.02) | 0.890 |

*Subgroup analysis with imputated BMI values, showcasing the pooled results (n=2544x5). ^a^Sensitivity analysis with imputed BMI values on patients with normal glucose metabolism, showcasing the pooled results (n=2544x5). The multivariable model was adjusted for PSA, ISUP scores, BMI, and comorbidities.*


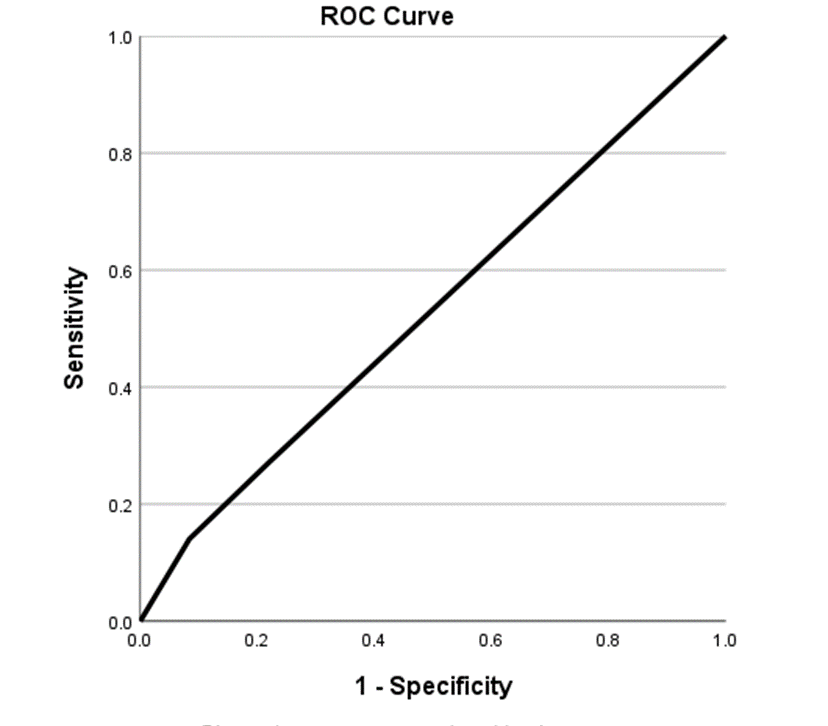

**Supplementary Figure I.** ROC Curve of univariable Cox regression model between testosterone groups and risk of mortality.


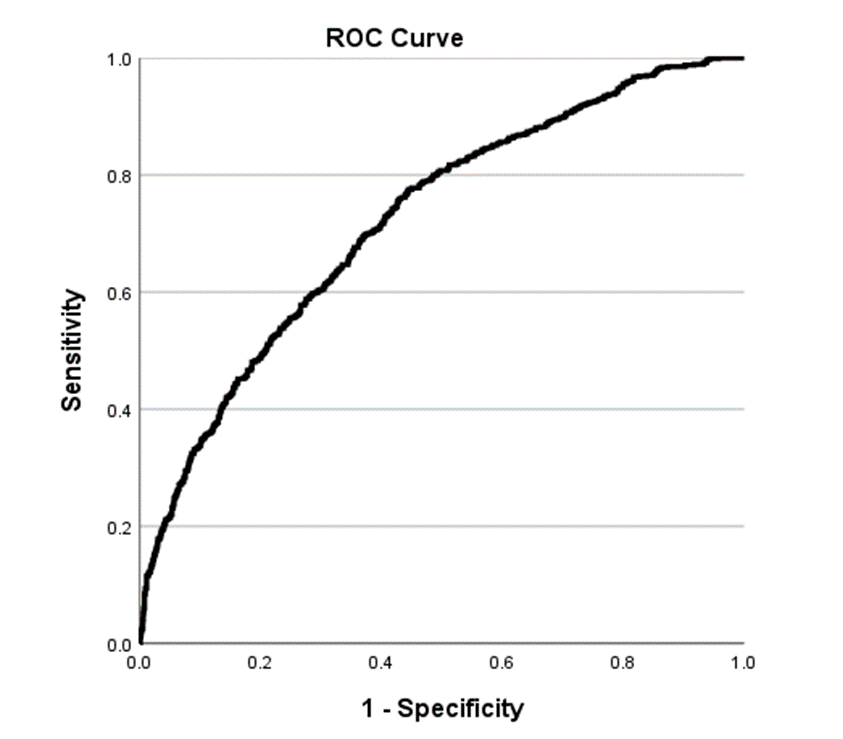

**Supplementary Figure II.** ROC Curve of multivariable Cox regression model between testosterone groups and risk of mortality. The multivariable model was adjusted for PSA, ISUP scores, age at PCa diagnosis, and comorbidities.
